# *Coxiella burnetii* as a model system for understanding host immune response against obligate intracellular, vacuolar pathogens

**DOI:** 10.1371/journal.ppat.1013071

**Published:** 2025-05-28

**Authors:** Akhila Sasikala, Chandhana Prakash, Mullai Valli Ramamoorthy, Sandhya Ganesan

**Affiliations:** 1 School of Biology, Indian Institute of Science Education and Research (IISER) Thiruvananthapuram, Thiruvananthapuram, Kerala, India; Duke University School of Medicine, UNITED STATES OF AMERICA

## Introduction

**Fig 1:**
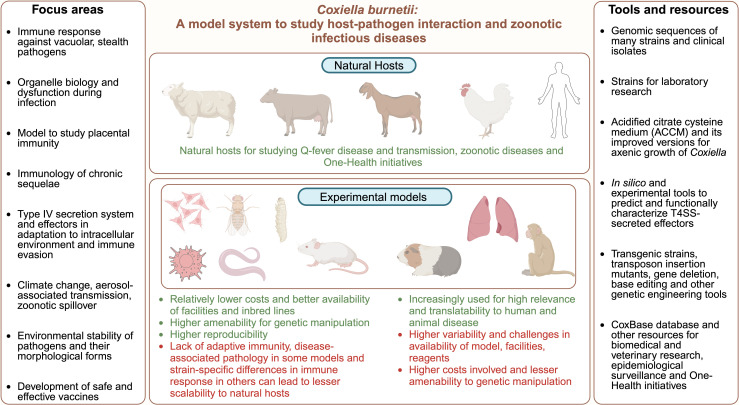
*Coxiella burnetii:* A model system to study host–pathogen interaction and zoonotic infectious diseases. Technological advances in the field of *C. burnetii* research, including the development of axenic media, availability of virulent and attenuated strains for laboratory research, *in silico* and experimental tools to predict and functionally characterize T4SS-secreted effectors (SecReT4, DeepSecE, T4SEpp) [92–94], enhanced genetic toolkit [21–24,88,89,95,96] and other resources (example: CoxBase) [97], as listed on the right panel, have facilitated better understanding of interaction between host and *C. burnetii* at a molecular, cellular, and organism level. Bottom panel depicts some of the experimental host systems employed, including, epithelial cells/ fibroblasts, macrophages of different animal origins, *Drosophila melanogaster*, *Caenorhabditis elegans*, *Galleria mellonella*, wildtype and SCID mice, guinea pigs, human *ex vivo* lung model, and non-human primates. These models are characterized by a spectrum of advantages and limitations, as highlighted by green and red text, respectively. These developments are expected to promote modeling of zoonotic disease biology in natural hosts (top panel) and research efforts in several key focus areas including dysregulation of lysosome biology by pathogens, placental immunity [98], effector functions, molecular mechanisms underlying chronic sequelae, environmental stability of *Coxiella*, impact of rising climate change, and vaccine development (left panel). Created with Biorender.com.

**Fig 2:**
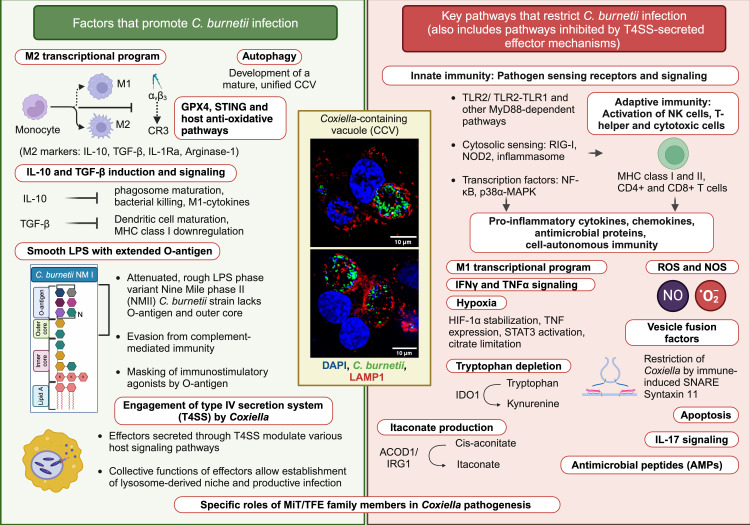
Factors promoting or restricting *Coxiella burnetii* infection. Development of bacterial replication-permissive, lysosome-derived compartment and intracellular bacterial proliferation (shown in the central boxed panel) is supported by the *Coxiella* T4SS and the translocation of numerous effectors into the host cell, M2 transcriptional program including upregulation of immunosuppressive cytokines TGF-β and IL-10, autophagy, and host anti-oxidative mechanisms. Further, the smooth LPS variants produced by the *C. burnetii* NMI strain mask the bacterial surface lipoproteins and prevent TLR2 activation, thus inhibiting pathogen sensing in the host and allowing the establishment of disease. At the same time, the host restricts *Coxiella* through activation of innate, adaptive, and cell-autonomous immune mechanisms including multiple pathogen recognition receptor-based pathways, activation of T and NK cells, secretion of pro-inflammatory cytokines, especially IFNγ, TNF, and IL-17, production of ROS and RNS, activation of the M1 program in macrophages, tryptophan depletion, production of itaconate, expression of STX11, antimicrobial peptide production, hypoxic conditions, and apoptosis. MiT/TFE family of transcription factors regulate *Coxiella* vacuole biogenesis and intracellular bacterial replication and the precise contribution of each, in promoting or restricting different stages of infection, requires further investigation. Central Boxed panel: Immunofluorescent images of TPA-differentiated THP1 cells infected with *C. burnetii* NMII for 3 days and fixed with 4% PFA. LAMP1, lysosome-associated membrane protein-1; TPA: 12-O-tetradecanoylphorbol-13-acetate; THP1: human monocyte cell line. Figure created with Biorender.com. Credit for immunofluorescent images in the central boxed panel: Ms. Amrita Bhattacharya, IISER Thiruvananthapuram.

Many intracellular bacterial pathogens have evolved to establish a secure vacuolar niche for replication in mammalian hosts through stealthy lifestyles and sophisticated mechanisms of virulence and adaptation. In particular, the bacterial pathogen *Coxiella burnetii* is increasingly gaining attention as an emerging zoonotic pathogen and an under-appreciated model to study host immune response against obligate intracellular pathogens. Here, we summarize the features that render *Coxiella* an excellent model to study basic, applied, and epidemiological questions in infectious disease research (Fig 1). Further, we provide a cheat sheet on how *Coxiella* has facilitated our understanding of innate, adaptive, and cell-autonomous immune responses to obligate intracellular pathogens (Fig 2).

## *Coxiella burnetii*: An emerging zoonotic pathogen and a great model system for biomedical research

*C. burnetii* was named in honor of Herald Rea Cox and Macfarlane Burnet, who initially identified and characterized this infectious agent along with Gordon Davis and Marvis Freeman [1,2]. *C. burnetii* belongs to the class Gammaproteobacteria, order Legionellales, and family Coxiellaceae. *C. burnetii* is a CDC select agent and NIAID priority pathogen due to its low infectious dose, high aerosol transmissibility, environmental stability, history of multiple outbreaks, and potential as an agent of bioterrorism [3,4]. The first and largest outbreak occurred in the Netherlands between 2007 and 2010, with >4000 human cases, presenting an unprecedented challenge to veterinary and public health [5]. Reservoirs of *Coxiella* include, but are not limited to, domestic animals (cattle, sheep, goats), wild birds, and rodents. *Coxiella* typically causes an acute febrile illness called “query- or Q-fever” and adversely affects animal reproductive fitness and productivity. *Coxiella* is transmitted from infected animals to humans primarily through aerosols, handling of infected tissues or birthing products, occupational exposure, and consumption of contaminated animal products*.* In humans, acute Q-fever is treatable with doxycycline. However, severe complications may also arise, including endocarditis, pneumonia, or a chronic fatigued condition called Q-fever fatigue syndrome [6]. While Q-fever is reportable in some countries (USA), it is not yet notifiable in many countries. The vaccine Q-vax (formalin-inactivated virulent strain) is licensed only in Australia and requires pre-screening due to the hypersensitivity of patients previously exposed to *Coxiella.* Coxevac, a veterinary vaccine, is available only in certain parts of Europe. Thus, there is a compelling need for a globally approved, safe vaccine.

The process of transition from a free-living/tick-associated ancestral lifestyle to an obligate intracellular environment is accompanied by a reduction in genome size, and loss/pseudogenization of many genes [7]. For instance, *C. burnetii*, containing a 2 Mb genome, has retained genes for heme, biotin, and LPS biosynthesis but is auxotrophic for almost 11 amino acids [7,8]. The most widely used strains for laboratory research are the Nine Mile Phase I (virulent and requires BSL-3 containment) and the attenuated phase variant, Nine Mile Phase II that was generated through continuous passage of NMI in yolk sacs and is suitable for use in BSL-2 laboratories [9]. NMI and NMII differ in their O-antigen and outer polysaccharide core but are indistinguishable in their lipid A and exhibit similar intracellular lifecycles [10–14] (Fig 2). In addition to phase variation, *C. burnetii* has two morphological forms, the spore-like small-cell variant (SCV) and the replicative large-cell variant (LCV) [15]. LCVs are metabolically more active and larger in size, whereas the SCVs exhibit greater stability to environmental stresses owing to a thick and remodeled peptidoglycan (PGN) layer [16,17].

At a single organism level, *Coxiella* is tropic to alveolar macrophages, phagocytic cells, placental, and heart tissues, but can infect multiple other cell types. Entry into host cells is followed by extensive fusion of the nascent vacuole with endolysosomal, autophagic, and secretory vesicles to generate a mature, acidic, spacious vacuole called *Coxiella*-containing vacuole (CCV) [18] (Fig 2). CCV biogenesis is mediated by bacterial proteins (called effectors) secreted through the Dot/Icm type IVB secretion system (T4SS) [19–24].

Thus, *Coxiella* (i) is evolutionarily adapted to and replicates in lysosomal-like environments, (ii) exhibits prolonged and persistent intracellular survival, and (iii) employs an extensive repertoire of effectors to intercept host vesicle traffic and manipulate host signaling. Here, we review the key determinants of protective host response against stealthy mammalian-adapted pathogens as learned from host and *Coxiella*-centric studies.

## Innate immunity

The two main virulence determinants of *Coxiella* are its LPS and the T4SS (Fig 2). Acute response is typically initiated by the detection of *Coxiella* by pattern-recognition receptors (PRRs) of the host cells, the production of pro-inflammatory cytokines (TNF, IL-1, and IL-18), the recruitment of monocytes to infection sites, and the formation of granulomas [25,26]. Despite being a Gram-negative pathogen, *Coxiella* LPS is tetra-acylated and is a weak activator of TLR4 [13,27]. TLR2, NOD2, and TLR1/TLR2 heterodimer also play an important role in cytokine production [13,27–31]. However, the precise mechanisms that allow cytosolic detection of the PGN derivatives of this vacuole-residing pathogen are unclear. The smooth LPS variant of NMI masks bacterial surface lipoproteins and evades detection by TLR2 [32] and complement-mediated killing [33]. It engages with ⍺_V_β_3_ integrin but inhibits CR3 activation, allowing higher survival inside monocytes compared to strains with rough LPS [34]. Other strains with (i) intermediate LPS length (NM Crazy), (ii) smooth LPS but deficient in Dot/Icm function (NMI *∆dot/icm*), (iii) natural NMII revertants with intermediate LPS (NMII-E), and (iv) smooth LPS but attenuated in animal models (Dugway) have also emerged as key resources for dissecting the distinct roles of LPS, its length, and the T4SS in driving innate immune responses [35–38]. T4SS-secreted effectors inhibit the activation of several innate immune signaling pathways including inflammasome, NF-κB, p38α-MAPK, IL-17, and RIG-I, to dampen cytokine response and promote bacterial replication [39–46]. *Coxiella* effectors also inhibit type I IFN production, which appears to play a dual role in tissue-specific models of infection [46,47].

Further, *Coxiella* induces the M2 transcriptional program and immunosuppressive cytokines, rendering monocytes and macrophages permissive to bacterial replication [48]. While TGF-β downregulates MHC-I and impairs dendritic cell activation, IL-10 signaling impairs phagosome maturation and bacterial killing, downregulates M1-cytokines including TNF and contributes to the development of chronic Q-fever [49–51].

## Adaptive immunity

Replication in a lysosomal-like vacuole allows the infected cell to present antigenic peptides and elicit T-helper cell immunity. Interestingly, both MHC-I and MHC-II are required for protective immunity, with one compensating in the other’s absence [52] (Fig 2). Consistent with this, mice lacking both CD4+ and CD8 + T cells succumb to *Coxiella* infection but are rescued by reconstitution of either of the T cell types [53,54]. MHC-I and CD8+ T cell-mediated immunity limits the spread of the bacterium, likely in part, through effector cytokines IFNγ and TNF [52]. However, the mechanisms that mediate MHC-I presentation of *Coxiella* antigens are not well understood. Interestingly, chronic Q fever is distinguished by high circulating levels of IFNγ and a high IFNγ/IL-2 ratio [55]. B cells limit tissue damage and disease severity but are not sufficient to control *Coxiella* [53,56]. However, antibody response is a useful serological diagnostic, since acute and chronic Q-fever are typically associated with predominantly high titer of antibodies against NM phase II and phase I, respectively [6].

## Cell-autonomous defense

Activation of the M1-program in infected cells inhibits intracellular *Coxiella* replication and maturation of the *Coxiella* vacuole (Fig 2). IFNγ, TNF, IL-17, MyD88-dependent and miRNA pathways restrict *Coxiella*, in part, by iNOS, NADPH, ACOD1, and IDO1 that mediate production of Nitric Oxide (NO), Hydrogen peroxide (H_2_O_2_), itaconate, tryptophan catabolism, apoptosis, and other unidentified mechanisms [27,45,57–66]. In hypoxic conditions, decreased availability of citrate/TCA metabolites also suppress intracellular *Coxiella* replication [67].

Microphthalmia/transcription factor E (MiT/TFE) family of proteins, in particular, TFEB and TFE3 appear to play a complex role in *Coxiella* infection. On one hand, overexpression of TFEB was observed to restrict *Coxiella* replication [68], and consequently, *Coxiella* inhibiting nuclear translocation of TFEB through T4SS-secreted effectors [69]. On the other hand, TFEB as well as TFE3 have been observed to be activated during infection [70,71] and double-deficiency was consistently observed to yield smaller CCVs [69–71]. However, TFEB/TFE3-double deficiency, depending on the genetic and cell type model, (KO mouse macrophages, siRNA/CRISPR-based KO HeLa) yielded either a concomitant decrease, increase or minimal impact on intracellular *Coxiella* replication [69–71]. It is possible that these seemingly divergent observations are likely because some TFEB/TFE3-regulated processes such as autophagy, lysosomal biogenesis are subverted by *Coxiella* for CCV expansion and fusogenicity, while others such as excessive CCV acidification and Cathepsin activity negatively influence bacterial replication inside the vacuole [68]. The specific contribution of TFEB, TFE3, MITF, and their temporal regulation during infection requires further investigation.

In addition to direct killing mechanisms, our work demonstrated that the expression of an immune-inducible SNARE, Syntaxin 11, which mediates vesicle fusion, also decreases bacterial replication and CCV expansion [72].

Conversely, host antioxidative pathways that reduce ROS levels, such as Glutathione Peroxidase 4 (GPX4) in humans, and a cytosolic sensor for cyclic dinucleotides (Stimulator of Interferon Genes, Sting) in *Drosophila*, promote *Coxiella* infection and increase host survival [73,74]. At least three T4SS-secreted effectors AnkG, CaeA, and CaeB inhibit intrinsic apoptosis in host cells, with CaeA also capable of inhibiting extrinsic apoptosis [75–79], and reviewed in [80]. Further, host pro-survival kinases Akt and Erk1/2 potentiate *Coxiella*’s anti-apoptotic activity [81]. These studies demonstrate that inhibition of programmed cell death is a key mechanism of intracellular adaptation of *Coxiella*. Typically, autophagy is associated with targeting pathogens for lysosomal degradation. However, autophagy and *ATG* gene functions are subverted by *Coxiella* for homotypic fusion of CCVs into a unified, spacious compartment [23,82].

Cells such as mast cells and neutrophils also contribute to bacterial control through secretion of antimicrobial peptides and control of bacterial dissemination [83,84]. Natural Killer (NK) cells limit intracellular bacterial replication and may protect from adverse histopathology, but release infectious *Coxiella* during degranulation [53,85].

## *Coxiella* research: Where are we headed?

We are bearing witness to remarkable developments in the field of *Coxiella* research, including the formulation and refinement of defined axenic media, the availability of genomic sequences, lab-adapted strains, and the development of genetic tools [8,86–89], making *Coxiella* increasingly accessible to investigate fundamental host-*Coxiella* interaction (Figs 1 and 2). Each experimental host model is associated with distinct advantages and limitations as highlighted in Fig 1 and this extensive review [90]. This expanding landscape of experimental systems and tools is expected to facilitate addressing several key questions in future, as listed in Fig 1 and S1 Box, address public, veterinary and One Health perspectives and better modeling of disease biology in natural hosts [91].

## Supporting information

S1 Box Key open questions regarding the immune mechanisms against *Coxiella burnetii.* The expanding knowledge, experimental toolkit and models available for *Coxiella* research (Fig 1) is expected to facilitate addressing several key questions in future, as listed in S1 Box. These questions represent broad areas of innate immunity, organelle biology and vesicle traffic, effector biology, stress response, infection and immunity-induced cell death, adaptive immunity, disease manifestation and strain/clinical variant-specific response and more. Created with Biorender.com.
(TIF)
