# Supplementary figures and images for: Coxiella burnetii as a model system for understanding host immune response against obligate intracellular, vacuolar pathogens
Source: PLoS Pathog. 2025 May 28;21(5):e1013071. doi: 10.1371/journal.ppat.1013071 (PMC12119012; doi:10.1371/journal.ppat.1013071)

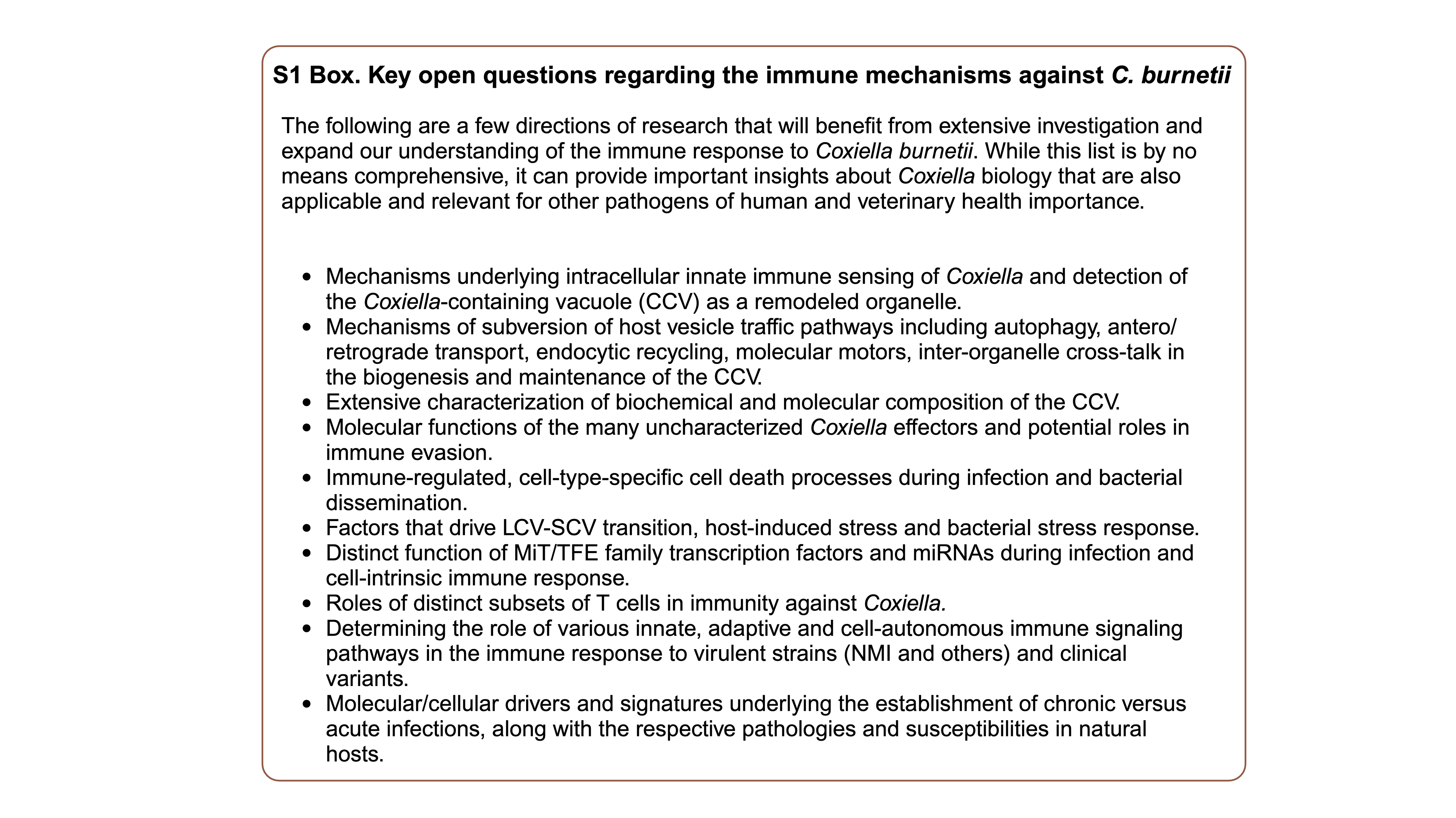

Supplement: S1 Box — The expanding knowledge, experimental toolkit and models available for Coxiella research (Fig 1) is expected to facilitate addressing several key questions in future, as listed in S1 Box. These questions represent broad areas of innate immunity, organelle biology and vesicle traffic, effector biology, stress response, infection and immunity-induced cell death, adaptive immunity, disease manifestation and strain/clinical variant-specific response and more. Created with Biorender.com. (TIF) [file ppat.1013071.s001.tif]
